# Design, reliability and construct validity of a Knowledge, Attitude and Practice questionnaire on personal use of antibiotics in Spain

**DOI:** 10.1038/s41598-020-77769-6

**Published:** 2020-11-26

**Authors:** Narmeen Mallah, Rubén Rodríguez-Cano, Adolfo Figueiras, Bahi Takkouche

**Affiliations:** 1 Department of Preventive Medicine, University of Santiago de Compostela, R/ San Francisco, s/n, 15782 Santiago de Compostela, Spain; 2 Centro de Investigación Biomédica en Red de Epidemiología y Salud Pública (CIBER-ESP), Madrid, Spain; 3 Department of Behavioral Science, The University of Texas MD Anderson Cancer Center, Houston, TX USA; 4 Health Research Institute of Santiago de Compostela (IDIS), Santiago de Compostela, Spain

**Keywords:** Public health, Epidemiology

## Abstract

Numerous questionnaires are available on Knowledge, Attitudes, and Practices (KAP) towards antibiotics' use by adults, but none of these questionnaires is fully validated. We undertook an exhaustive literature review to design a comprehensive KAP questionnaire concerning the personal use of antibiotics in Galicia, North Spain. The Item Content Validity Index (I-CVI) and modified Kappa statistic (K*), confirmed the content validity of the questions (0.78 ≤ I-CVI ≤ 1.00 and 0.78 ≤ K* ≤ 1.00). The S-CVI statistic showed the content validity of the scale (S-CVI/Ave: 0.95). Following face validity and pilot testing, the Test–Retest Reliability in a sample of 145 adults confirmed the reliability of the questions. We carried out Confirmatory Factor Analysis using cross loadings and modification indices to choose the most adequate model in data collected from 844 adults. We estimated the indicators of model fit and demonstrated that the selected model has a good to excellent fit, thus establishing the construct validity. The final version of the questionnaire was highly accepted by the general adult population as reflected by the response rate (95.85%) and the low percentage of unanswered questions (0.4–2.7%). Our fully validated questionnaire could prove useful for research as it permits generating high quality data and reducing measurement error.

## Introduction

Antibiotic resistance remains a global public health threat, despite the exerted regional and international efforts to defeat this problem. In Europe, the burden of infections due to antibiotic-resistant bacteria is comparable to that of influenza, tuberculosis and HIV/AIDS combined^1^. Each year, Europe records 670,000 new infections due to resistance and 33,000 deaths as a direct consequence of these infections. A recent report from the Center of Disease Control showed that the burden of antibiotic resistance remains important in the United States, with 2.8 million antibiotic-resistant infections and more than 35,000 related deaths every year^2^. It is foreseen that the situation by 2050 will deteriorate even more, with 10 million attributable annual deaths and a cumulative cost of 100 trillion USD, if no proactive solutions are found to slow down the expansion of antibiotic resistance^3^.

Health strategies to tackle antibiotic resistance were already defined by the Word Health Organization and encompass interventions that are oriented to health and research centers as well as to community settings^4^. Increasing awareness and changing behaviors towards a proper use of antibiotics are essential elements in the community intervention package^4^. In this context, intervention programs to improve the use of antibiotics apply a specific questionnaire pre- and post-intervention in order to measure changes in Knowledge, Attitudes and Practices (KAP) in the target population. KAP modeled questionnaires are instruments that assess the following three dimensions: Knowledge (what the respondents know about antibiotics), Attitude (what the respondents think or believe about antibiotics) and Practice (what they do regarding antibiotics)^5^.

As data obtained from KAP studies are fundamental in assessing the need, planning and implementing public health programs, it is of paramount importance to use reliable and valid instruments in order to ensure a good research quality^6^. Nonetheless, despite the long-standing recognition of antibiotic resistance, the literature revealed that to-date, no fully validated KAP questionnaire is available for antibiotics’ use by the general population^7^. A recent systematic review of surveys about knowledge and/or attitudes towards antibiotic use by the general population reported that 11 studies tested the validity and the reliability of the used questionnaire^8^, but the validity of these questionnaires was only partially assessed and limited to face and/or content validity. Other studies claimed using “previously similar validated questionnaires”^9^, nonetheless the cited studies were not found to include validated questionnaires. Alumran and colleagues developed and validated the construct of a questionnaire about the parents’ perceptions on the use of antibiotics for children with upper respiratory tract infections^10^. However, this questionnaire cannot be applied in a straightforward fashion to assess knowledge and attitudes of adults concerning their own use of antibiotics, as parents are usually more judicious with antibiotics’ use for children than for themselves^11^.

Accordingly, to fill this gap, we aimed in the present study to design and validate a questionnaire for the assessment of Knowledge and Attitude related to Practices of personal use of antibiotics in the general population. We describe the development process of this questionnaire and report the assessment of its face, content and construct validity. We also examine the questionnaire reliability, responsiveness, and acceptability in North Spain.

## Results

### Content validity

**Figure 1:**
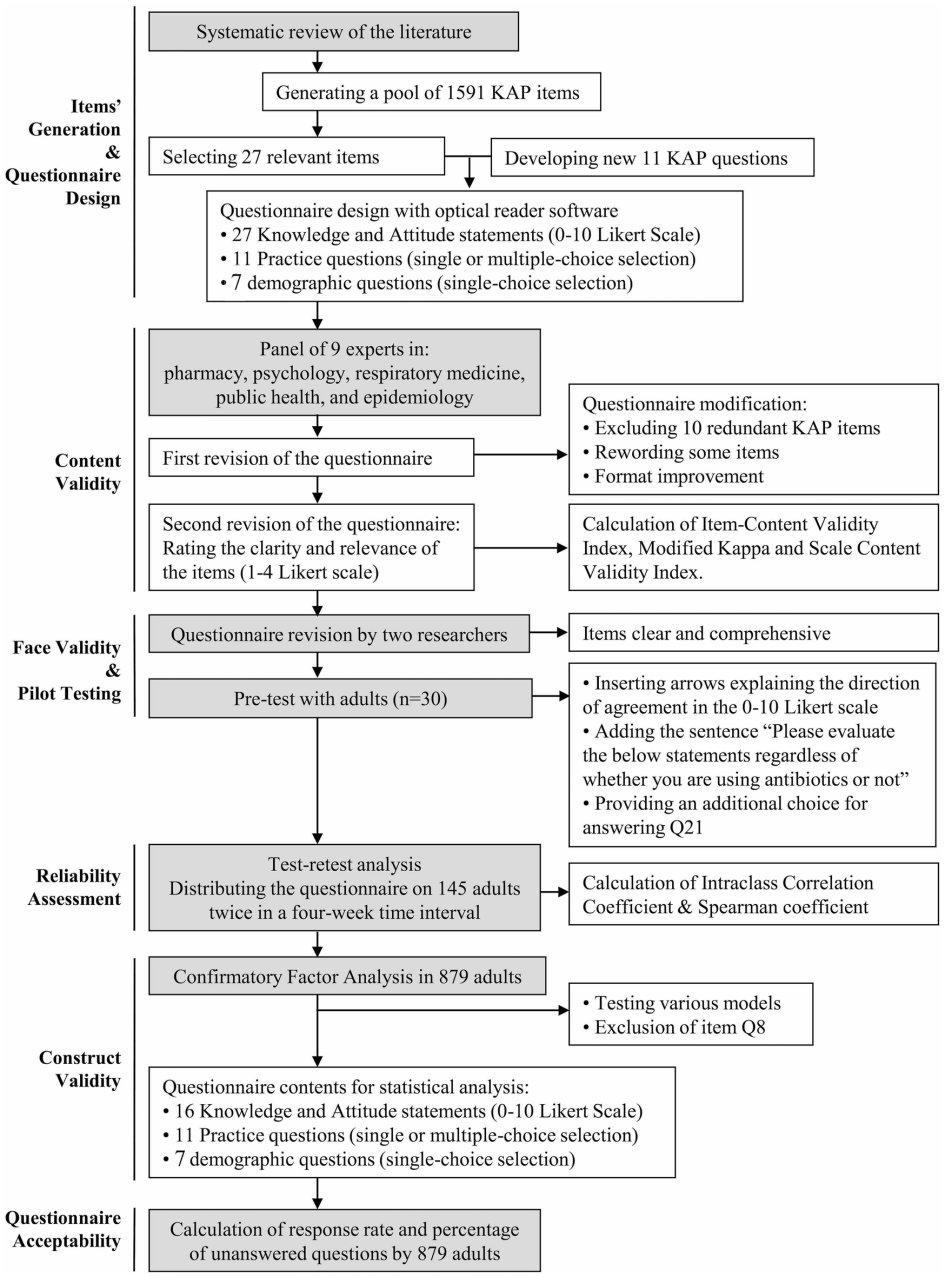
Flow diagram describing the steps followed to develop and validate the KAP questionnaire.

The process of items’ selection for retaining in the questionnaire to be validated is summarized in Fig. 1. The Item-Content Validity Index (I-CVI) estimations for the 28 KAP items that were retained after the initial evaluation of the panelists ranged between 0.78 and 1.00, indicating that these items were considered clear, understandable, and relevant to the questionnaire. For all items, modified Kappa (K^*^) values were excellent (> 0.75), revealing that the agreement between experts was not due to chance. Scale Content Validity Index (S-CVI/Ave) was 0.95, confirming the content validity of the scale.

### Face validity and pilot testing

All the 30 adults who participated in the pilot testing answered the questionnaire in its totality. Two participants reported that they misunderstood 0 and 10 being the lowest and highest levels of agreement. We, therefore, added an additional indication using arrows to help the participants remember the direction of agreement. We also provided an answered example (“practicing sports benefits health”) to facilitate the understanding of the 0–10 Likert Scale concept. One participant declared that it was not clear whether the Knowledge and Attitudes items should be answered in case the respondent did not use antibiotics. Therefore, we added this statement “Please evaluate the below statements REGARDLESS of whether you are using antibiotics OR NOT”. For the question, “The last time you had to take antibiotics, did you complete the course of treatment?”, the participants suggested adding one additional answer (“still using them”). The questionnaire took 8–10 min to be completed, and the participants showed satisfaction about the questionnaire length.

### Test–retest reliability

Out of the 145 adults invited to participate in the test–retest reliability test, 140 answered the questionnaire in the two occasions. The Intraclass Correlation Coefficient (ICC) assessment showed that the reliability was acceptable for 4 items (0.40 ≤ ICC ≤ 0.58), good for 9 items (0.60 ≤ ICC ≤ 0.70), and excellent for an item (ICC = 0.82) (Table 1). Three items (Q5, Q10 and Q11) showed ICC values below 0.4 (Table 1). Since low ICC could be due to lack of sample heterogeneity for these items, we calculated their Spearman´s correlation coefficient in order to explore further their reliability. Spearman coefficient showed that answers in the first and second rounds are weakly correlated for Q5 (Spearman regression coefficient, r_s_ = 0.193; p = 0.022) and moderately correlated for Q10 (r_s_ = 0.433; p < 0.0001) and Q11 (r_s_ = 0.405; p < 0.0001).

**Table 1:** Test–retest reliability assessment of Knowledge and Attitudes items of the questionnaire.

Item	N	ICC (95% CI)
Q1. Antibiotics are effective against viruses	137	0.82 (0.75, 0.87)
Q2. When I get a cold, I take antibiotics to help me feel better faster	140	0.62 (0.48, 0.73)
Q3. If I feel better after a few days, I sometimes stop taking my antibiotics before completing the course of treatment	139	0.40 (0.17, 0.57)
Q4. I expect my doctor to prescribe antibiotics if I suffer from common cold or flu symptoms	138	0.68 (0.55, 0.77)
Q5. It is good to be able to get antibiotics from relatives or friends without having to see a medical doctor	140	0.06 (− 0.32, 0.32)
Q6. When I have a sore throat, I prefer to use an antibiotic	138	0.62 (0.46, 0.73)
Q7. Each type of infection needs a different antibiotic	138	0.67 (0.54, 0.77)
Q8. Antibiotics can kill the bacteria that normally live on the skin and in the gut	130	0.67 (0.54, 0.77)
Q9. If I feel side effects during a course of treatment of antibiotics, I should stop taking them as soon as possible	136	0.60 (0.44, 0.72)
Q10. I take the antibiotics according to the doctor’s instructions	139	0.36 (0.10, 0.54)
Q11. If antibiotics are consumed in excess, they will not work when they are really needed	137	0.19 (0.14, 0.42)
Q12. I prefer to keep antibiotics at home in case there is a need for them later	139	0.58 (0.41, 0.70)
Q13. I trust the doctor’s decision if s/he decides to prescribe or not prescribe antibiotics	138	0.50 (0.29, 0.64)
Q14. If I believe that I need an antibiotic and the doctor did not prescribe it, I will get it at the pharmacy without a prescription	138	0.50 (0.29, 0.64)
Q15. Doctors often explain clearly to the patient the reasons for prescribing or not prescribing antibiotics	134	0.70 (0.58, 0.79)
Q16. Doctors often explain clearly to the patient the instructions for the use of antibiotics	138	0.65 (0.51, 0.75)
Q17. When you buy antibiotics, the pharmacist tells you about the importance of correct therapeutic compliance/adherence	139	0.63 (0.48, 0.74)

*N* number of participants who answered the corresponding item in the two-time occasions, *ICC* intra-class correlation coefficient.

### Construct validity

We explored the construct validity by distributing the initial 17 items of the Knowledge and Attitude construct into 2 dimensions: Knowledge about antibiotics (Knowledge), and Attitude towards antibiotics (Attitude), based on theoretical grounds and then by confirming the structure of the 2-factors model (Model 0) using Confirmatory Factor Analysis (CFA). This model showed an unacceptable model fit as reflected by the goodness of fit indicators (Table 2).

**Table 2:** Comparison of the goodness of fit parameters between models.

Indicator	Model 0	Model 1.0	Model 1.1
χ^2^	1037.074	580.45	248.49
df	118	116	94
p	< 0.0001	< 0.0001	< 0.0001
RSMEA (90% CI)	0.096 (0.091, 0.102)	0.069 (0.063, 0.075)	0.044 (0.038, 0.051)
CFI	0.55	0.77	0.92
TLI	0.49	0.74	0.90
AIC	67,594.035	67,141.414	62,873.317
BIC	67,840.296	67,397.146	63,147.993
aBIC*	67,675.160	67,225.659	62,963.803
SRMR	0.088	0.073	0.047

Model 0 encompassed two factors (Knowledge and Attitude), Model 1.0 consisted of three factors (Knowledge, Attitude-Personal and Attitude-Healthcare provider) and Model 1.1 involved the same factors as Model 1.0 but excluding Q8.

*χ*^*2*^ Chi-square value, *df* Degree of Freedom, *p* p-value (Chi-square), *RSMEA* Root Mean Squared Error Approximation, *CFI* Comparative Fit Index, *TLI* Tucker-Lewis Index, *AIC* Akaike Information Criterion, *BIC* Bayesian Information Criterion, *aBIC* sample-size adjusted BIC, *SRMR* Standardized Root Mean Square Residual.

In Model 0, Q8 did not load significantly in the Knowledge factor, and the items “Q10, Q13, Q15, Q16, Q17” loaded negatively in the Attitude factor. These five attitude items dealt with the patient-health care provider relationship and therefore they were attributed to a new factor (Attitude-Healthcare provider).

The new model (Model 1.0) included “Q1, Q2, Q4, Q6, Q7, Q8 and Q11” in the Knowledge factor; “Q3, Q5, Q9, Q12, Q14” in Attitude-Personal factor and “Q10, Q13, Q15, Q16, Q17” in Attitude-Healthcare provider factor. Model 1.0 showed better fit than Model 0, however it still was not acceptable (Table 2).

**Figure 2:**
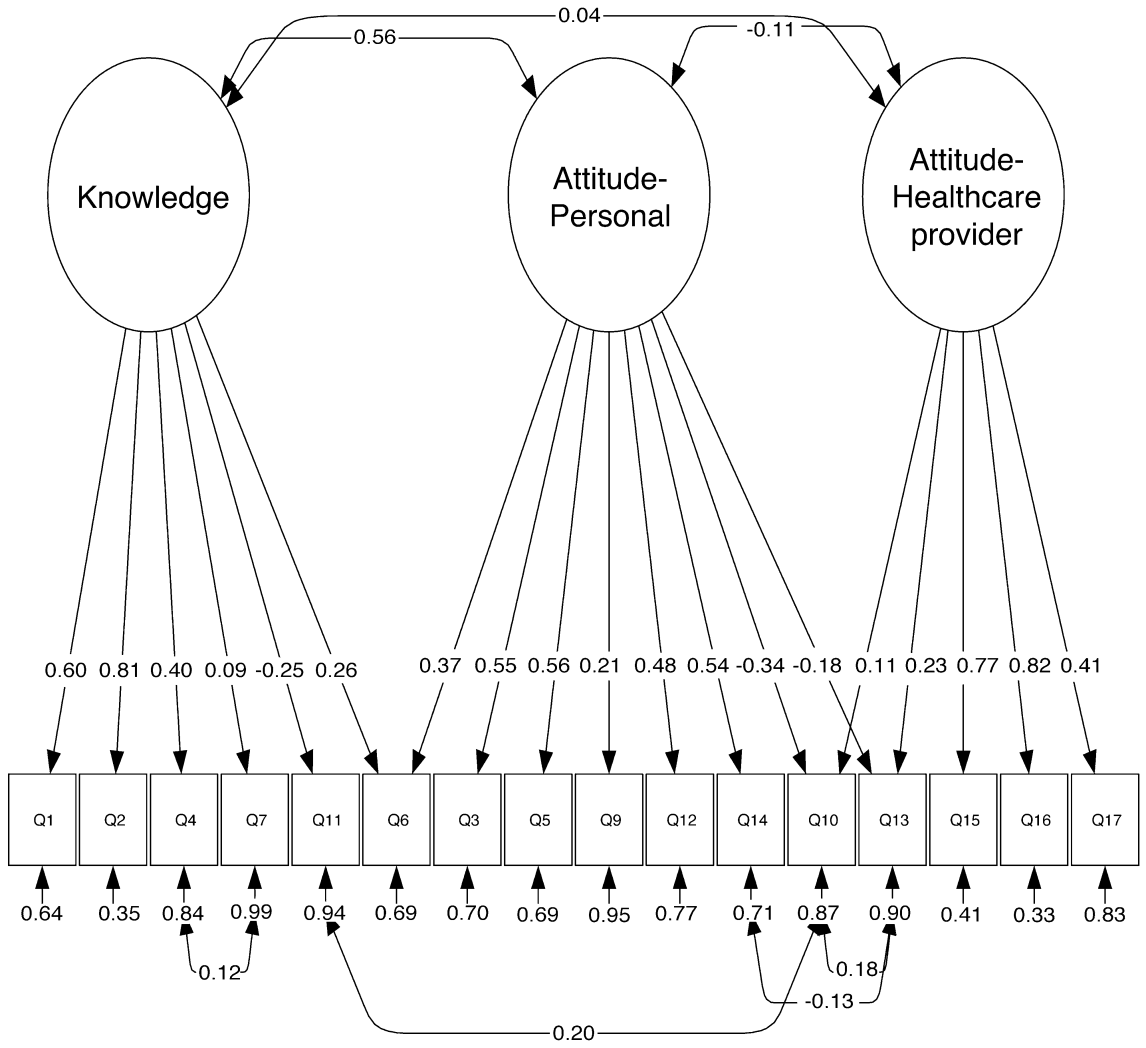
Representation of the model selected by CFA analysis (Model 1.1). Each of the three factors (Knowledge, Attitude-Personal and Attitude-Healthcare provider) is represented with its corresponding standardized items loadings and their residuals. “Knowledge” includes items the explore the knowledge of the adults towards antibiotics. “Attitude-Personal” encompasses statements about attitudes towards the personal use of antibiotics. “Attitude-Healthcare provider” involves phrases about the patient-healthcare provider relationship with respect to antibiotics. The double-sided arrows represent correlations between the variables. Q1–Q7 and Q9–Q17 are items of the Knowledge and Attitude construct (Q8 was deleted in a previous step). The single headed arrows represent the correlation of the items and their respective factors.

Therefore, we made various iterations based on cross-loadings, modification indices and analyzed correlations between items' residuals in order to improve the goodness of fit. Accordingly, we eliminated Q8 “Antibiotics can kill the bacteria that normally live on the skin and in the gut” from Model 1.0, and named the modified model “Model 1.1”. Table 3 summarizes the loadings of the 16 retained items on their corresponding factor. Significant correlations were observed between the residuals of Q10 and Q13 (r = 0.18, p < 0.0001), Q11 and Q10 (r = 0.20, p-value < 0.0001), Q13 and Q14 (r = − 0.13, p-value = 0.001) and Q4 and Q7 (r  = 0.12, p-value = 0.001) (Fig. 2).

**Table 3:** Factors loadings and standard errors from the three-factors model (Model 1.1).

Item	Loading estimate	Standard error	P-value	Standard loading estimate
**Knowledge**				
Q1. Antibiotics are effective against viruses	1.65	0.09	< 0.0001	0.60
Q2. When I get a cold, I take antibiotics to help me feel better faster	1.46	0.08	< 0.0001	0.81
Q4. I expect my doctor to prescribe antibiotics if I suffer from common cold or flu symptoms	1.22	0.09	< 0.0001	0.40
Q6. When I have a sore throat, I prefer to use an antibiotic	0.49	0.09	< 0.0001	0.26
Q7. Each type of infection needs a different antibiotic	0.12	0.08	0.014	0.09
Q11. If antibiotics are consumed in excess, they will not work when they are really needed	− 0.38	0.06	< 0.0001	− 0.25
**Attitude-personal**				
Q3. If I feel better after a few days, I sometimes stop taking my antibiotics before completing the course of treatment	1.55	0.09	< 0.0001	0.55
Q5. It is good to be able to get antibiotics from relatives or friends without having to see a medical doctor	0.66	0.05	< 0.0001	0.56
Q6. When I have a sore throat, I prefer to use an antibiotic	0.84	0.12	< 0.0001	0.37
Q9. If I feel side effects during a course of treatment of antibiotics, I should stop taking them as soon as possible	0.62	0.11	< 0.0001	0.21
Q10. I take the antibiotics according to the doctor’s instructions	− 0.40	0.05	< 0.0001	− 0.39
Q12. I prefer to keep antibiotics at home in case there is a need for them later	1.28	0.09	< 0.0001	0.48
Q13. I trust the doctor’s decision if s/he decides to prescribe or not prescribe antibiotics	− 0.34	0.08	< 0.0001	− 0.18
Q14. If I believe that I need an antibiotic and the doctor did not prescribe it, I will get it at the pharmacy without a prescription	0.90	0.06	< 0.0001	0.54
**Attitude-healthcare provider**				
Q10. I take the antibiotics according to the doctor’s instructions	0.12	0.04	0.004	0.11
Q13. I trust the doctor’s decision if s/he decides to prescribe or not prescribe antibiotics	0.39	0.06	< 0.0001	0.23
Q15. Doctors often explain clearly to the patient the reasons for prescribing or not prescribing antibiotics	1.76	0.08	< 0.0001	0.77
Q16. Doctors often explain clearly to the patient the instructions for the use of antibiotics	1.69	0.08	< 0.0001	0.82
Q17. When you buy antibiotics, the pharmacist tells you about the importance of correct therapeutic compliance/adherence	1.05	0.07	< 0.0001	0.41

The indicators of the goodness of fit assessment of the Model 1.1 showed an adequate fit. (Table 2). The *χ*^2^ difference test between Model 1.0 and Model 1.1 showed that they are statistically different (Δ *χ*^2^ = 331.97, Δdf = 22, p = 0.0001) (Table 2). Accordingly, we adopted Model 1.1 for this questionnaire.

Knowledge was significantly positively correlated with Attitude-Personal factor (r = 0.56, p < 0.0001), whereas it was not correlated with Attitude-Healthcare provider (r = 0.04, p = 0.417). Attitude-Personal and Attitude-Healthcare provider were weakly negatively correlated (r = − 0.11, p = 0.023) (Fig. 1).

### Final version of the questionnaire

According to the findings from the validation steps mentioned above, we provide the final version of the questionnaire which consists of three blocks (see Supplementary Information S1). The first block encompasses the 16 retained items after the CFA analysis and that underlie 3 factors: Knowledge about antibiotics, Attitude towards the personal use of antibiotics and Attitude towards health care providers. The second block includes a series of 11 questions that intend to measure the different practices of personal misuse of antibiotics. The third block entails the demographic characteristics and consists of seven questions. The questions of the second and the third blocks are responded by selecting one or several answers from a list of possible answers, except for “age” which is introduced as a number by the participants.

### Questionnaire overall reliability

The overall reliability was reflected by the Cronbach's alpha of 0.62, which is deemed acceptable given that our questionnaire assesses distinct dimensions uncorrelated with each other.

### Items and questionnaire acceptability

844 out of 879 participants accepted answering the questionnaire, yielding a participation rate of 95.85%. The percentage of missing or blank answers was between 0.4% and 2.7%, revealing a high item-response rate. These figures indicate high acceptability of the questionnaire by the population.

## Discussion

To-date none of the studies that measured the association of Knowledge and Attitudes with Practices of use of antibiotics in the general population applied a fully validated questionnaire^8^. Therefore, to fill this gap, we designed and validated the psychometric properties of a KAP questionnaire about antibiotics’ use by the adult general population. The content and the scale validity indices confirmed the content validity of our questionnaire. Moreover, the test–retest reliability and the confirmatory factor analysis proved the reliability and the construct validity of the questionnaire. Importantly, the questionnaire was accepted by the general population as reflected by the high response rate and the low percentage of unanswered questions.

The *availability* of a reliable and construct-valid instrument is fundamental for epidemiological studies that aim to measure associations between Knowledge, Attitudes, and Practices toward the personal use of antibiotics, as the use of a non-validated questionnaire may induce measurement error in the exposure and the outcome. In particular, the availability of a validated KAP questionnaire is crucial for the design of interventions aimed at improving the Knowledge and modifying Attitudes and Practices towards a proper use of antibiotics.

Knowledge and Attitudes are considered stable variables that are not likely to be changed rapidly. The test–retest *reliability* of these questions showed that they generate reproducible results, except for the item Q5 “It is good to be able to get antibiotics from relatives or friends without having to see a medical doctor”. The low ICC for Q5 could be due to the fact that the sample distribution was concentrated in the extreme lower end of the agreement scale^12^, where the overwhelming majority of the population answered by 0 (totally disagree) in the two test rounds. Moreover, an extremely large proportion of participants showed disagreement with the statement Q5. Accordingly, item Q5 is likely to be reliable and should be retained in the questionnaire.

The *construct validity* assessment showed that the adopted model has a good to excellent fit. The logical and the theoretical distribution of the questions across the three factors, Knowledge, Attitude-Personal and Attitude-Healthcare provider, in Model 1.1 supported further the adequacy of the model^13^. In our questionnaire, some items loaded in more than one factor (cross-load). This was expected since our instrument encompasses a multidimensional construct^14^. Knowledge factor correlated significantly with Attitudes towards the personal use of antibiotics (Attitude-Personal). Knowledge and Attitude-Personal include item Q6 “When I have a sore throat, I prefer to use an antibiotic” that loaded significantly on both factors, explaining further the existing association. Such findings were expected, as personal Attitudes toward the use of antibiotics in case of sore throat infections are in part driven by patients’ knowledge on this topic. On the other side, Knowledge was not correlated with Attitudes towards health-care provider (Attitude-Healthcare provider), revealing that the items designed to measure participants’ Knowledge about antibiotics are not related to those intended to explore the relation between patients and healthcare providers. This confirms that the questions included in each of these two factors are specific and measure unique and unrelated dimensions. Our CFA results indicate that including the factor Attitude-Healthcare provider in the questionnaire is essential as various reports highlighted on the influence of healthcare provider-patient relationship on proper therapeutic compliance^15,16^, and in specific with respect to the proper use of antibiotics^17,18^. Consequently, KAP questionnaires should not be limited to knowledge and personal attitudes only, but should also examine attitudes towards healthcare-providers. In addition, a negative and weak correlation existed between Attitude-Personal and Attitude-Healthcare provider, suggesting that individuals who tend to have higher agreement with Attitude-Healthcare provider statements are also more prone to disagree or to agree to a lesser extent with the items included in Attitude-Personal factor. This demonstrates that trust and communication between patients and their healthcare providers have a substantial impact on patients’ attitudes toward antibiotics. Explaining the motives for prescribing or not prescribing antibiotics and giving instructions of their use by the physicians to the patients is associated with lower odds of antibiotics’ misuse^18^. Testing these associations in other populations would further validate our findings.

In general, an assessment of past intake of antibiotics relies on the memory of the participants. Therefore, to decrease the risk of recall bias we have included a time limit in the design of the “Practice” questions by asking about the use in the past two months. The questionnaire encompasses 11 questions that are elaborated to determine any aspect of misuse. Moreover, the provided choices of answers were based on an extensive literature review to include any possible answer, and therefore avoid leaving questions unanswered.

The questionnaire was designed to measure the participants’ Knowledge and Attitudes regardless of their consumption of antibiotics in the last 2 months. Therefore, our questionnaire could prove useful in obtaining data both from users and non-users of antibiotics, which represent a crucial issue in epidemiologic studies involving Knowledge, Attitudes and Practices in antibiotic use.

The high *acceptability* of the questionnaire by the general population reflects the feasibility of its application in general population settings. In fact, the time taken to answer our questionnaire was within the ideal range (10 min), which therefore aided in increasing the response rate^19^. Questionnaires with a long list of questions negatively influence the participation rate and the quality of data^20^. Another factor that could have enhanced the response rate is the fact of being issued from a research and academic institution^21^. The same questionnaire was translated forward and backward into English, Arabic and French in a previous study that targeted the general population^18^. The translated versions were also pilot tested. This favors the application of the questionnaire in non-English speaking populations.

Our study has an important limitation. In epidemiologic studies, an important step in the validation process involves comparing the results obtained from the questionnaire being validated to a superior method, deemed “gold standard”. However, to the best of our knowledge, to date, a gold standard to assess the proper use or the misuse of antibiotics does not exist and therefore, our instrument could not be compared against any previous reference method. Due to this limitation, we consider that the present questionnaire is reliable and has construct validity, but future research is needed to provide a gold standard for KAP questionnaire about antibiotics. Another limitation of our validation study is that the construct was validated in the Spanish population only, therefore our questionnaire needs to be further tested in different settings and populations.

## Conclusion

This study presents a step forward towards the validation of a knowledge, attitude, and practice questionnaire about the personal use of antibiotics. Moreover, taking into consideration the inconsistent reporting of validation methodologies across studies and the abuse of the term validation, as well as the exhaustive review of the methodology carried out in the current manuscript, we believe that this study would help validating KAP pharmacologic studies on other drugs than antibiotics.

## Methods

### Items’ generation

We comprehensively reviewed the literature to identify published KAP questionnaires about the personal use of antibiotics in the general population. We applied the following search syntax in Medline from inception until September 2018: (("Anti-Bacterial Agents"[Mesh] OR "Anti-Bacterial Agents" [Pharmacological Action]) AND ("Surveys and Questionnaires"[Mesh]) AND ("Attitude to Health"[Mesh] OR "Health Knowledge, Attitudes, Practice"[Mesh] OR "Knowledge"[Mesh] OR beliefs OR perception OR "Health Behavior"[Mesh] OR "Awareness"[Mesh]) AND (misuse or overuse or use or abuse)). We also searched conference papers in the Conference Proceedings Citation Index-Science (CPCI-S) as well as the reference list of relevant studies. In addition, we reviewed reports about the determinants of self-medication with antibiotics as well as aspects of medicine’s misuse. Subsequently, we generated a pool of 1591 published items (questions or statements) about 3 dimensions: knowledge, attitudes, or practices towards antibiotics. After removing duplicated or very similar questions, we selected 27 items based on their relevancy to the topic, and tailored their wordings as needed. We also created 11 additional KAP questions in order to draft a comprehensive questionnaire on the mentioned dimensions. We included seven questions about demographic characteristics. The 45-item questionnaire was originally written in English and then forward and backward translated to Spanish/Galician language by bilingual researchers. The translated version of the questionnaire was then reviewed by a native language specialist. The questionnaire was designed using OMR Remark Office software (Remark Office OMR 2014, version 9.2.0.20, GRAVIC, PA, USA).

### Content validity

The content validity of the questionnaire is an assessment of the adequateness and the comprehensiveness of the items of the questionnaire to measure the target construct and is routinely performed by a panel of 3–10 experts^22,23^.

Our panel of experts consisted of 9 members with experience in questionnaire design and who were specialized in at least one of the following fields: pharmacy, psychology, respiratory medicine, public health, and epidemiology. At first, we provided the panel of experts with the 45-item questionnaire and collected their feedback about the items’ clarity, relevance, and ease of understanding as well as the comprehensiveness of the questionnaire. The experts received the Spanish/Galician version of the questionnaire, as Spanish/Galician is their native language. They were also requested to identify deficient areas, suggest any additional potentially relevant item and/or possible answer, and make suggestions for improvement. The questionnaire was then modified by discarding any unnecessary item, rephrasing any ambiguous question, and making the necessary changes in the format. The modified questionnaire was circulated among the panelists for a second evaluation. At this stage, each of the nine experts, evaluated the content of the questionnaire by rating each item, using a 1–4 Likert scale. 1 and 4 represented the lowest and the highest levels of clarity and relevance, respectively. We calculated the Item Content Validity Index (I-CVI) by dividing the number of experts rating that item ≥ 3 by the total number of experts. As our panel consisted of nine experts, items with I-CVI ≥ 0.78 were retained in the questionnaire^24^.

We then calculated the Scale Content Validity Index (S-CVI/Ave) which represents the average of I-CVIs of all items of the scale. S-CVI/Ave > 0.90 is considered to reveal a content-valid scale^24^.

To take into account the agreement by chance between experts we estimated a modified Kappa (k*)^25^. The probability of agreement by chance (P_c_) was calculated using the formula: P_c_ = (N/A (N − A) × 0.5^N^, where N is the total number of experts, A is the number of experts that rated the item by ≥ 3. K^*^ is calculated as follows: Κ^*^ = (I-CVI − P_c_)/(1 − P_c_)^25^.

### Face validity and pilot testing

Face validity entails an examination of the questionnaire by the research group in order to determine whether the included items are appropriate and relevant and whether the questionnaire measures what it is intended to be measured; i.e. Knowledge, Attitudes and Practices towards antibiotics^22^. Therefore, subsequent to content validity, two members of the group (N.M. and B.T.) subjectively checked the face validity of the questionnaire by reviewing the clarity and the completeness of the questions to measure the target outcome.

The questionnaire was then tested in a sample of 30 adults from the general population who were not related to the medical field. We asked the participants to provide feedback about the clarity and the understandability of the questions, the questionnaire design, the ease of answering and finally on the time taken to answer the questionnaire.

### Test–retest reliability

Reliability is concerned with measurement error and it reflects the stability of the measurement process over time^22,26^. Stability is routinely evaluated through test–retest procedure. It involves administering the same questionnaire to the same participants on two occasions, provided that the measured characteristic does not change during the testing period^26^. Since Knowledge and Attitudes are considered stable characteristics over time, we examined the stability of these domains by conducting a test–retest reliability assessment in a sample of 145 adults. The participants were randomly recruited from the administrative staff at the University of Santiago de Compostela, Spain. Participants were unrelated to the health or medical fields. We administered the same questionnaire to the same participants within a 4-week time interval. The participants were informed about the study objective and they agreed to answer the questionnaire on the two occasions.

We assessed the reliability by calculating the Intraclass Correlation Coefficients (ICCs) with their 95% Confidence Interval (CI) relative to the average measure of the two-way mixed-effects model as recommended for test–retest settings^27^. Items with ICC ≥ 0.4 were considered reliable^28^.

### Construct validity

Construct validity is an assessment of the extent to which a questionnaire measures a target construct, i.e. Knowledge and Attitudes. Factorial validity represents an empirical assessment of the construct validity by applying factor analysis statistical models. A factor is a combination of items that are thought to measure the same dimension or trait (such as knowledge)^22^. This step is carried out when the construct of the questionnaire is intended to measure more than one dimension. The Knowledge and Attitude construct was designed to comprise two dimensions of 17 items. These variables were measured in a 0–10 Likert Scale.

We carried out a Confirmatory Factor Analysis (CFA) to test the construct validity of the questionnaire. CFA assesses the relationships between the items and their corresponding factor. We distributed the questionnaire in a population of 879 adult individuals from the general population. The participants consisted of subjects accompanying a next-of-kin to primary care consultations at the University Hospital of Santiago de Compostela, Spain. All subjects visiting the consultations during the recruitment period that took place between May and December 2019 were contacted. They were informed that the questions are about personal use of antibiotics, and not about the use in the offspring, in order to avoid any misunderstanding.

We structured the factorial model by assigning each item to its specific dimension (factor) according to theoretical grounds^29–32^. We started by distributing the 17 items of the construct into two factors. Items that tested the knowledge about antibiotics’ role and specificity were assigned to the Knowledge factor. Items that inspected the agreement of the participants about certain attitudes towards the use of antibiotics and patient-health care provider relationship were placed in the Attitude factor. Subsequently we tested the correlation of the items with their corresponding factors.

We explored the normality of the distribution of each item included in the construct and then undertook CFA using the Maximum Likelihood Robust estimation method. Missing data were handled by applying Full Information Maximum Likelihood (FIML). The factors were standardized by constraining them to a mean of 0 and to a variance of 1. To improve the fit of the model, we inspected standardized residual correlations between items and applied modification indices method in order to better select the items to be added to a factor^13,33^. Standardized factor loadings represent the correlation between an item and its corresponding factor. We ran three CFA and compared the fit of the models against each other.

We assessed the goodness of fit of the models using the following statistics: RSMEA, CFI, TLI and SRMR. We also compared AIC, BIC and aBIC. AIC indicates the relative amount of information lost by a model. Lower AIC values indicate higher quality of the model. BIC is an indicator similar to AIC, however it penalizes the model more than AIC^34^.

RMSEA values are considered “excellent” if < 0.06 and acceptable if RMSEA < 0.08. CFI and TLI values ≥ 0.95 indicate excellent fit and values between 0.90 and 0.94 indicate acceptable fit. SRMR values < 0.08 indicate acceptable fit^35^.

### Questionnaire overall reliability

We calculated Cronbach’s coefficient alpha to check the overall reliability of the questionnaire using data collected from the 844 adult individuals^26^. A reliability index ≥ 0.6 is considered acceptable^36,37^.

### Items and questionnaire acceptability

We explored the acceptability of the questionnaire in the cohort of 879 individuals by calculating the response rate, i.e. the percentage of individuals who accepted answering the questionnaire. We also inspected the item-response rate by computing the percentage of missing data for each item^38–42^.

All statistical analyses were carried out using IBM SPSS 20.0. CFA was analyzed with R (version 4.0.0)^43^, and R package: lavaan (version 0.6–6)^44^.

The flow diagram of the full validation procedure is summarized in Fig. 1.

### Ethics

Our study was approved by the ethics committee of the University of Santiago de Compostela (R00002, No. 2019/179). It was also authorized by the Spanish Agency for Medication and Healthcare Products (AEMPS, Reference AFG-ANT-2018-01). The study was conducted in compliance with the general requirements of the ethics committee and with the General Data Protection Regulation (Regulation (EU) 2016/679 and Organic Law 3/2018). Written informed consent form was obtained from the participants and the data were anonymized before analysis.

## Supplementary information

Supplementary Information.

## Data Availability

The dataset generated and analysed during the current study is available in the (FigShare) repository, (https://figshare.com/s/d8bbd91b657d9a468aaa).
