# Supplementary material for: Design, reliability and construct validity of a Knowledge, Attitude and Practice questionnaire on personal use of antibiotics in Spain
Source: Sci Rep. 2020 Nov 26;10:20668. doi: 10.1038/s41598-020-77769-6 (PMC7693171; doi:10.1038/s41598-020-77769-6)
Supplement: Supplementary file 1 — Supplementary Information. [file 41598_2020_77769_MOESM1_ESM.pdf]

Design, reliability and construct validity of a Knowledge, Attitude and Practice questionnaire on personal use of antibiotics in Spain

Narmeen Mallah[1,2], Rubén Rodríguez-Cano[3], Adolfo Figueiras[1,2,4], Bahi Takkouche[1,2,4*]

[1]Department of Preventive Medicine, University of Santiago de Compostela, Santiago de Compostela, Spain

[2]Centro de Investigación Biomédica en Red de Epidemiología y Salud Pública (CIBER-ESP), Madrid, Spain

[3]Department of Behavioral Science, The University of Texas MD Anderson Cancer Center, Houston, TX; USA.

[4]Health Research Institute of Santiago de Compostela (IDIS), Santiago de Compostela, Spain

[*]**: Corresponding author:** Bahi Takkouche, Department of Preventive Medicine, University of Santiago de Compostela, Santiago de Compostela, Spain, R/ San Francisco, s/n, 15782, [bahi.takkouche@usc.es], +34-881-812-268

KNOWLEDGE, ATTITUDES AND PRACTICES TOWARDS THE PERSONAL USE OF ANTIBIOTICS

Code of the questionnaire: ____________________ Date: ________________________

To choose an answer, please fill in <u>COMPLETELY</u> the corresponding circle(s): CORRECTLY answered: ● INCORRECTLY answered: ⊠ ∅

Please answer on the following questions about **ANTIBIOTICS** (Amoxicillin, Cefixime, Ciprofloxacin, Levofloxacin, Penicillin, etc.)

On a scale of 0 to 10 points, choose a number representing your level of agreement on the statements below. "0" (zero) means you totally disagree while "10" means that you agree completely.

Please evaluate the below statements **REGARDLESS** of whether you are currently using *antibiotics* **OR NOT**

Example: Practicing sports benefits health (0) (1) (2) (3) (4) (5) (6) (7) ● (9) (10)

Statement	← Disagree — Agree →
**1. *Antibiotics* are effective against viruses**	(0) (1) (2) (3) (4) (5) (6) (7) (8) (9) (10)
**2. When I get a cold, I take *antibiotics* to help me feel better faster**	(0) (1) (2) (3) (4) (5) (6) (7) (8) (9) (10)
**3. If I feel better after a few days, I sometimes stop taking my *antibiotics* before completing the course of treatment**	(0) (1) (2) (3) (4) (5) (6) (7) (8) (9) (10)
**4. I expect my doctor to prescribe *antibiotics* if I suffer from common cold or flu symptoms**	(0) (1) (2) (3) (4) (5) (6) (7) (8) (9) (10)
**5. It is good to be able to get *antibiotics* from relatives or friends without having to see a medical doctor**	(0) (1) (2) (3) (4) (5) (6) (7) (8) (9) (10)
**6. When I have a sore throat, I prefer to use an *antibiotic***	(0) (1) (2) (3) (4) (5) (6) (7) (8) (9) (10)
**7. Each type of infection needs a different *antibiotic***	(0) (1) (2) (3) (4) (5) (6) (7) (8) (9) (10)
**8. If I feel side effects during a course of treatment of *antibiotics*, I should stop taking them as soon as possible**	(0) (1) (2) (3) (4) (5) (6) (7) (8) (9) (10)
**9. I take the *antibiotics* according to the doctor's instructions**	(0) (1) (2) (3) (4) (5) (6) (7) (8) (9) (10)
**10. If *antibiotics* are consumed in excess, they will not work when they are really needed**	(0) (1) (2) (3) (4) (5) (6) (7) (8) (9) (10)
**11. I prefer to keep *antibiotics* at home in case there is a need for them later**	(0) (1) (2) (3) (4) (5) (6) (7) (8) (9) (10)
**12. I trust the doctor's decision if s/he decides to prescribe or not prescribe *antibiotics***	(0) (1) (2) (3) (4) (5) (6) (7) (8) (9) (10)
**13. If I believe that I need an *antibiotic* and the doctor did not prescribe it, I will get it at the pharmacy without a prescription**	(0) (1) (2) (3) (4) (5) (6) (7) (8) (9) (10)
**14. Doctors often explain clearly to the patient the reasons for prescribing or not prescribing *antibiotics***	(0) (1) (2) (3) (4) (5) (6) (7) (8) (9) (10)
**15. Doctors often explain clearly to the patient the instructions for the use of *antibiotics***	(0) (1) (2) (3) (4) (5) (6) (7) (8) (9) (10)
**16. When you buy *antibiotics*, the pharmacist tells you about the importance of correct therapeutic compliance/adherence**	(0) (1) (2) (3) (4) (5) (6) (7) (8) (9) (10)

Code of the questionnaire: ____________________ **Date:** ________________________

17. In the past two months, did you take antibiotics? ○ Yes (please move to question 18) ○ No (please move to question 28)

Answer the following questions about your consumption of ANTIBIOTICS <u>IN THE PAST TWO MONTHS</u>

18. How long was the duration of your last treatment with *antibiotics*?

Number of Days ☐☐

Number of Months ☐☐

19. Who prescribed or recommended you the use of *antibiotics*? (You can choose more than one answer)

- ○ The doctor
- ○ Friends
- ○ The family
- ○ The pharmacist
- ○ I had them at home

20. The last time you had to take *antibiotics*, did you complete the course of treatment?

- ○ Yes
- ○ No
- ○ Still using them

21. What did you do with the *antibiotics* that were left unused? (You can choose more than one answer)

- ○ I kept them to be used the next time I am sick
- ○ I disposed of them in the garbage or sewage system
- ○ I gave them to someone
- ○ I didn´t have any remaining *antibiotics*

22. The last time you had to take *antibiotics*, did you forget to take any of the doses?

- ○ Never (please move to question 24)
- ○ Sometimes (please move to question 23)
- ○ Often (please move to question 23)

23. What did you do when you skipped a dose of your *antibiotics*?

- ○ I continued the following doses normally
- ○ I doubled/increased the following dose
- ○ I took it as soon as I remembered

24. The last time you had to take *antibiotics*, did you change the dose on your own (without medical advice)?

- ○ Never (please move to question 28)
- ○ Sometimes (please move to question 25)
- ○ Often (please move to question 25)

25. What did you do when you changed your dose of *antibiotics* on your own (without medical advice)?

- ○ I took more of it (please answer question 26 and then 28)
- ○ I took less of it (please move to question 27)
- ○ Sometimes I took more, and sometimes I took less of it (please answer questions 26 and 27)

26. Why did you increase the dose of *antibiotics* on your own (without medical advice)? (You can choose more than one answer)

- ○ I forgot to take the previous dose
- ○ I felt very sick
- ○ I didn't feel a notable improvement
- ○ I felt better but wanted to improve even more

27. Why did you reduce the dose of *antibiotics* on your own (without medical advice)? (You can choose more than one answer)

- ○ I was worried about the medication's side effects
- ○ I was tired, and I forgot
- ○ I felt that I was taking too many medicines
- ○ I was in a hurry and I forgot
- ○ I don't like to take medicines at night
- ○ I was feeling better

KNOWLEDGE, ATTITUDES AND PRACTICES TOWARDS THE PERSONAL USE OF ANTIBIOTICS

Code of the questionnaire: ____________________ Date: ______________________

General Demographic Characteristics

28. Indicate your gender

- ○ Male
- ○ Female

29. Indicate your age ___________

30. What is your highest educational level?

- ○ I did not go to school
- ○ Primary
- ○ Secondary
- ○ University

31. Are you currently working?

- ○ Yes
- ○ No

32. How many members live in your house, including you?

- ○ 2
- ○ 3-4
- ○ 5-6
- ○ More than 6

33. Do you consult a doctor when you are sick?

- ○ Never
- ○ Rarely
- ○ Sometimes
- ○ Always

34. Have you ever received a medical prescription over the phone?

- ○ Yes
- ○ No

Thank you very much for your time and collaboration!
